# Unusually High Thermopower in Molecular Junctions from Molecularly Induced Quantized States in Their Semimetal Leads

**DOI:** 10.1021/acs.nanolett.4c05852

**Published:** 2025-02-07

**Authors:** Mor Cohen Jungerman, Shachar Shmueli, Pini Shekhter, Yoram Selzer

**Affiliations:** † Department of Chemical Physics, School of Chemistry, Tel Aviv University, Tel Aviv 69978, Israel; ‡ The Tel Aviv Center for Nanoscience and Nanotechnology, Tel Aviv 69978, Israel

**Keywords:** thermovoltage, Seebeck, space-charge region, quantum confinement, molecular junctions

## Abstract

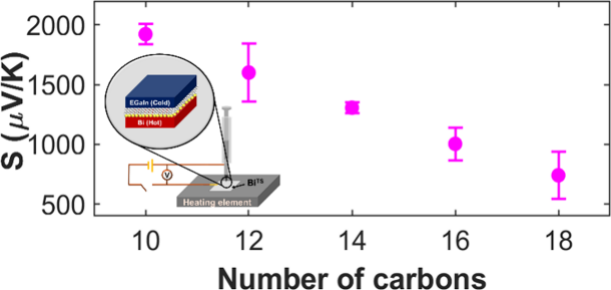

The efficiency of a thermoelectric (TE) device depends on the extent to which its electron/hole transport symmetry at the Fermi level is broken. This requirement makes molecular junctions promising for TE applications as their transmission characteristics are highly nonlinear. Yet, in the absence of an efficient method to tune the position of the Fermi level within their transmission landscape, the typical Seebeck values of metal–molecules–metal junctions are |*S*| ≤ 100 μV/K, while considering their electrical and thermal conductance, it should be |*S*| ≥ 1 mV/K to be relevant for applications. Here, we report metal–molecules–semimetal junctions with |*S*| in the required mV/K range. This is achieved by molecularly induced quantized two-dimensional (2D) interfacial states within the semimetal that result in nonlinear features in their transmission properties. The importance of the presented approach goes beyond TE applications as it demonstrates a novel strategy to form and tune 2D interfacial layers within bulk materials by molecular monolayers.

Within the current broad interest in thermal transport at the nanoscale and quantum thermodynamics, thermoelectric (TE) systems are of particular importance as they provide direct thermal-to-electrical power conversion.^1−12^ In a two-terminal device a necessary condition for thermoelectricity in the linear regime (at zero bias and under a small temperature gradient, Δ*T*) is breaking of the electron–hole transport symmetry in the vicinity of the Fermi level.^13−22^ The essential role of the transmission  in the realization of this requirement is quantified for ballistic devices by the expression of the Seebeck coefficient using the Landauer formalism:
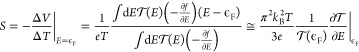
1where *ϵ*_F_ and *f* are the Fermi energy and distribution, respectively, *k*_B_ is the Boltzmann constant, and *e* is the charge of an electron. Since both ∂*f*/∂*E* and (*E*–*ϵ*_*F*_) are symmetric in terms of electron/hole transport at the Fermi level, it is the energy dependency of , which can break this symmetry and maximize *|S|*. This prerequisite to control  for better performance makes molecular junctions attractive as TE devices due to their nonlinear properties and the notion that they can readily be tuned and optimized for better efficiency by modifying the structure of the molecules. Most of the effort in this route has been focusing on molecules that feature destructive quantum interference in their transport properties, which indeed results in highly nonlinear  behavior.^23−34^ Yet, as the Mott formula on the right side of eq 1 emphasizes, high values of *|S|* are expected only when the nonlinear feature occurs at the Fermi energy. The practical difficulty to realize this essential energy alignment has so far resulted, except in very few sporadic exceptions,^26,35^ in reported *|S|* values that are <100 μV/K. While, based on the electrical and thermal conductance values of typical molecular junctions, the necessary value of *|S|* should be in the mV/K regime to make molecular TE devices suitable for (most likely low power) applications.^36^ Although, potentially, the necessary Fermi level tuning can be achieved in single molecule junctions by electrostatic gating,^14,35^ the low tunability and reproducibility of the gating effect, along with their low fabrication yield and stability, make these junctions unlikely relevant for applications, although still an excellent spectroscopy tool to study the fundamentals of nanoscale thermal transport.^35^ We postulate that molecular TE devices are more likely to be two-terminal junctions and for the purpose of better stability in the form of molecular ensemble junctions (MEJs).

Here, we wish to present an alternative approach to indue highly nonlinear transport properties in molecular junctions at the Fermi level by considering the following expression for :^37^
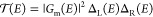
2where *G*_m_ is the Green function of the molecule and Δ_L/R_ are the spectral densities of the leads, which depend on their interfacial density of states (DOS) and carry information about their coupling to the molecules. Thus, while previous methods focused on changing *G*_m_ by changing the molecular structure, our approach is based on molecularly induced and controlled modification of Δ in one of the leads, which for this purpose needs to be a semimetal. We note that a similar reasoning has already been discussed theoretically by considering molecular junctions with a semiconductor as one of the leads^38,39^ and also in other studies^40,41^ that demonstrate that processes such as metallic alloying and underpotential deposition of metallic layers can also be used to alter the interfacial DOS within the leads of junctions and in turn affect their conductance and TE properties. Yet, all these studies do not report values of *|S|* in the mV/K regime and still require a capability of Fermi level tuning.

To better demonstrate the merits of Δ manipulation, we use metal-molecules-semimetal MEJs based on alkane chains. Junctions with these molecules with only metal leads are typically characterized by |*S*| ≤ 10 μV/K due to the large energy gap between their highest occupied and lowest unoccupied molecular orbitals (HOMO–LUMO gap), that results in gradual and “boring”  behavior in the vicinity of the Fermi level (see eq 1).^42^ Here, by exploiting the capability to molecularly tweak Δ in the same energy range with “boring”  behavior, we report |*S*| > 1 mV/K, i.e., an increase of the Seebeck by 3 orders of magnitude surpassing the threshold for applications.^36^

The semimetal lead in all used MEJs is bismuth (Bi) oriented in the (111) direction, which is the naturally formed orientation of Bi on amorphous substrates (see SI for details). The choice of Bi is rationalized by several of its attributes. Its weak electrostatic screening (relative permittivity of *ϵ*_Bi_ = 100)^43,44^ and small effective mass of its electrons at the Fermi level moving perpendicular to the interface (*m*_⊥_ = 0.05*m*_0_, where *m*_0_ is the mass of a free electron)^45−47^ are both essential for the alteration of the DOS in the form of two-dimensional (2D) quantized states at the semimetal-molecules interface.

The third attribute is the fact that molecular monolayers can readily be assembled on Bi surfaces.^48−50^ The formation of such layers has also been shown to effectively strip-off the native oxide of Bi during the assembly process.^49^ The fourth attribute is the very small DOS (∼4.2 × 10^–6^ states eV^–1^ atom^–1^) of Bi in the vicinity of the Fermi level (in Au, for example, it is ∼0.1 states eV^–1^ atom^–1^), which eliminates masking effects such as metal-induced gap states (MIGS).^44,48^

The measurement setup, shown schematically in Figure 1a, facilitates conductance and thermovoltage measurements^49,50^ at room temperature under ambient conditions (see also SI). The formed MEJs have a Eutectic Gallium–Indium (EGaIn) drop as a top soft contact and a 60 nm film of Bi as a bottom lead.^50^ Further details on the measurements can be found in the SI. All measured junctions are based on the following alkane amines: H_2_N–(CH_2_)_*n*−1_–CH_3_, with *n* = 10,12,14,16,18, abbreviated below as NC_10_, NC_12_, NC_14_, NC_16_ and NC_18_, respectively.

**Figure 1:**
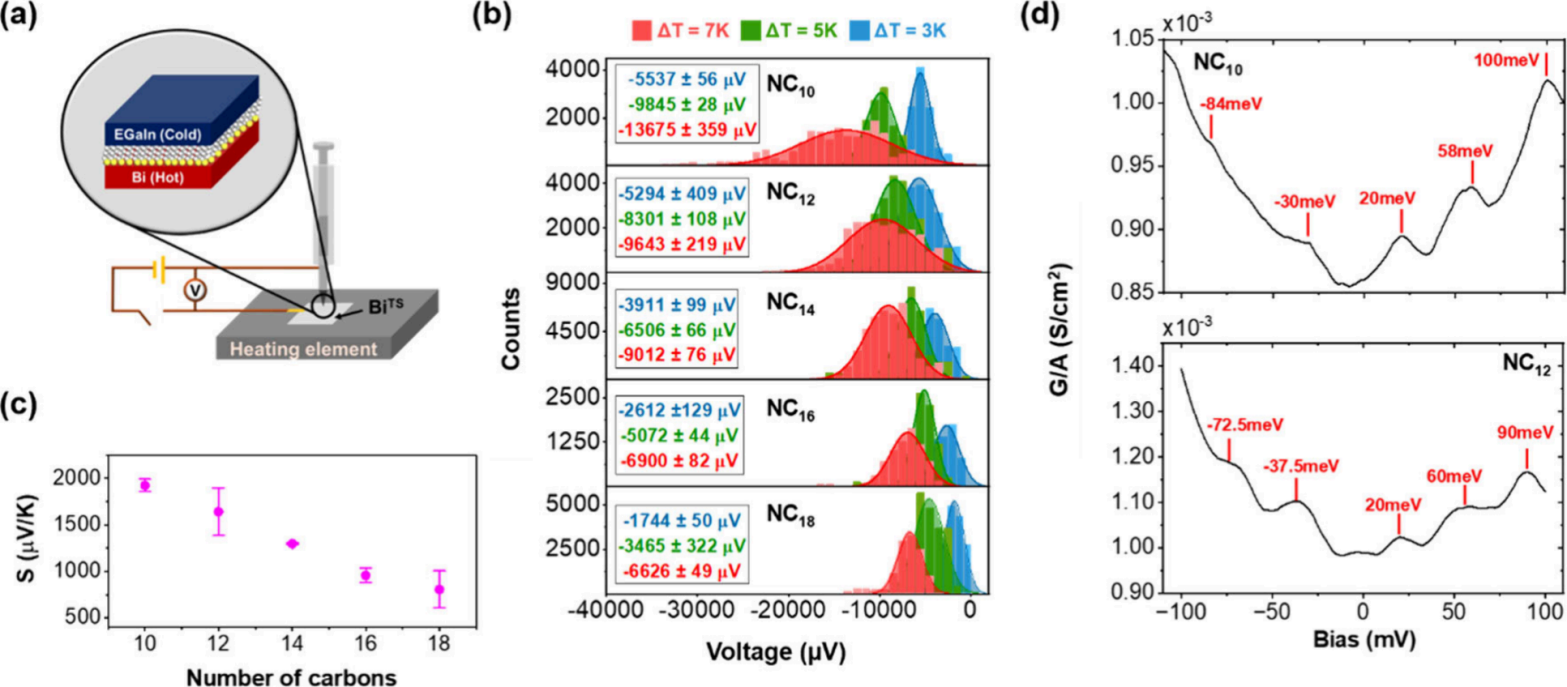
Experimental setup and main results. (a) A schematic presentation of an EGaIn/molecules/Bi junction with the basic circuit to enable both conductance and thermovoltage measurements. (b) Histograms of thermovoltage measurements at three different values of temperature gradients for each type of molecule. (c) Seebeck values as a function of molecular length. (d) Conductance measurements, performed by a lock-in technique at room temperature, of NC_10_ and NC_12_ junctions. Additional curves for the same molecules appear in the SI.

Figure 1b plots thermovoltage histograms of the different junctions under temperature gradients of Δ*T* = 3, 5, and 7 K and the resulting Seebeck coefficients calculated from these measurements as a function of *n* are summarized in Figure 1c. Two important observations can be made by examining these results. First, is the value range of |*S|* ∼ 0.8–2 mV/K, which is uniquely high and is the main result of this research. Second, is the fact that |*S*| appears to decrease as a function of *n*. This is an important observation, since due to the large HOMO–LUMO gap of alkane chains their low bias electron transport mechanism is by off-resonance (superexchange) tunneling,^48,51,52^ which should be accompanied by an |S| that increases with *n* according to |*S|* ∝ *n*/(*E*_Molecular_ – *E*_F_), where *E*_Molecular_ is either *E*_HOMO_ or *E*_LUMO_ depending which molecular level dominates the transport process^51,52^ (see further discussion in the SI). Indeed, this expected behavior has been observed in junctions with alkanethiols adsorbed on Bi.^50^ As discussed below, the apparent decrease of |*S*| with *n* is a proof for (length-dependent) molecularly induced changes in Δ within the Bi that masks the expected |*S|* ∝ *n*/(*E*_Molecular_ – *E*_F_) behavior that is basically derived if one only considers the *G*_*m*_ argument in eq 2 under a wide band approximation.

Another finding that support induced changes in the DOS (in Δ) is shown in Figure 1d, which depicts conductance-voltage curves measured by a lock-in detection scheme of NC_10_, NC_12_ junctions at room temperature. Such measured peaks, within a bias range of ±0.1 V have never been observed with junctions of alkane chains. As their width is ∼20 meV, they cannot be associated with inelastic tunneling processes due to vibrational modes, as then their width should have been ∼5.4*k*_B_*T* > 100 meV (where *k*_B_ is the Boltzmann constant).^53^

A quantitative understanding of all observations necessitates first to examine the band alignment process that is taking place in these junctions upon their formation. For this purpose, ultraviolet photoemission spectroscopy (UPS) of the different layers adsorbed on Bi has been performed. Comparison between the onset signals of spectra with and without the adsorbed molecules establish the energy location of the HOMO of the molecular layers (Figure 2a), which appears to progressively slightly shift to lower values below the Fermi level as the molecules become longer.

**Figure 2:**
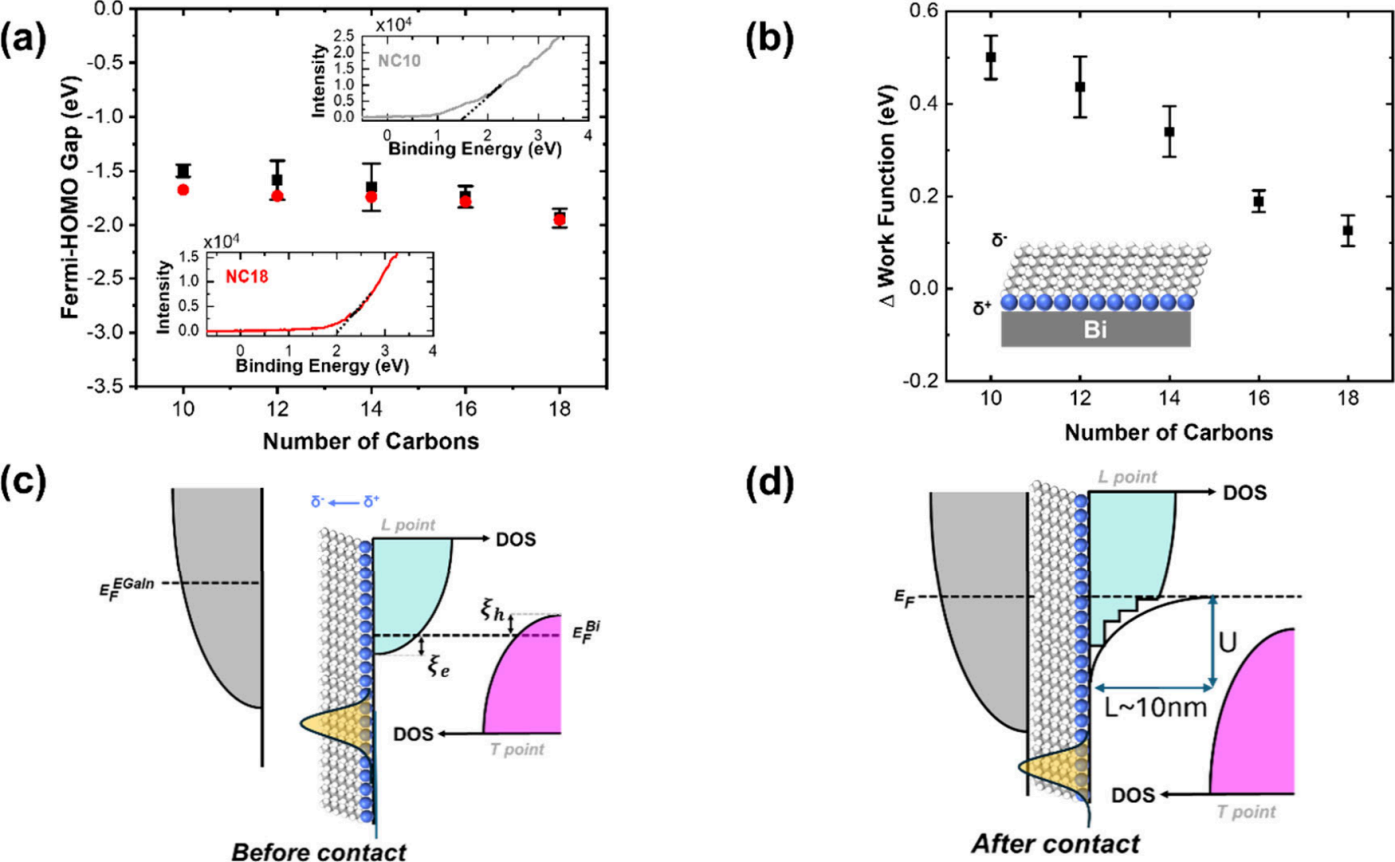
Band alignment processes. (a) In black is the energy position of the HOMO levels of the different molecules assembled on Bi, as determined by UPS. In red is their calculated new position as a result of band alignment within the junctions (see text for details). The insets show examples of how the position of the HOMO levels were determined from the UPS onset signal for NC_10_ and NC_18_. (b) Change in the work function of Bi (ΔWF) as a result of the assembly of different amino-alkane monolayers on its surface. The inset depicts schematically the molecular dipole formed on the surface. (c and d) The band alignment in the junctions. The DOS within the leads (gray for the EGaIn and magenta/cyan for Bi) are shown schematically with a general behavior of DOS, where *E* is versus the edge of each band. (c) Before contact. The dipole of the adsorbed layer of amino alkanes increases the work function of Bi and therefore in the scheme versus the vacuum level (not drawn) the Fermi level of Bi (*E*_F_^Bi^) is lower than the Fermi level of the EGaIn (*E*_F_^GaIn^). (d) After contact, the Fermi levels are aligned, and an accumulation layer is formed within the Bi. The DOS of the electron band (cyan) becomes quantized within the potential well that is formed at the interface (see text for details). The valence band (magenta) is shifted down and away from the Fermi energy. The HOMO level responsible for the transport through the molecules is shown (in orange) both in (c) and (d) to emphasize that the quantized states are formed near the Fermi level and deep within the HOMO–LUMO gap of the molecules. For clarity the LUMO is not drawn.

Figure 2b shows the change in the work function of Bi, ΔWF, induced by the different monolayers. In terms of magnitude, the absolute values of ΔWF are in the same range as those previously measured for monolayers of alkanethiols on Bi.^49,50^ However, here they are opposite in sign (ΔWF > 0) and the work function of Bi increases upon the assembly of alkane amines. As a result, while in EGaIn–alkanethiols–Bi junctions, a built-in potential has been shown to be formed within the Bi lead upon the formation of these junctions and in a form of a depletion layer,^49,50^ here, since the work function of the metal (EGaIn, 4.15 eV) is smaller than all modified Bi films (4.3 eV + ΔWF), the space-charge region within the Bi is expected to be in a form of an accumulation layer, i.e., as a potential well at the interface.

This expected band alignment process is shown schematically in Figures 2c and 2d. For the calculation of *U*, the depth of the interfacial potential wells and their extent, *L*, into the bulk, the band structure of Bi needs to be considered. The valence band (shown in magenta in Figure 2c) overlaps with the conduction band (cyan), each with the Fermi level very close to the band edge, with the respective values of: *ξ*_h_ = 12 meV and *ξ*_e_ = 22 meV.^46,47^ The small Fermi energies correspond to a density of charge carriers (*n*_0_ = 3.5 × 10^17^ cm^–3^) that is 5 orders of magnitude smaller than in typical metals such as Au or Ag (∼10^22^ cm^–3^). This low density of carriers and the accompanying small electrostatic screening enable the formation of the built-in potential, *u*, within the Bi side of the junctions.^49,50^ Details of the calculation of this potential, *u*(*x*), can be found in the SI. Figure 3a shows that for all molecular layers, *u* increases from its bottom value *U* below the Fermi level along the *x* direction into the bulk in the following way *u*(*x*) = −*Ue*^–*x*/*L*^ with *L* calculated to be ∼100 Å. This value of *L* is of particular importance since Bi structures and films with critical dimensions of this order have been reported to have quantized electronic levels.^54−56^ Yet, since here the Bi lead is essentially a bulk structure, quantization of states commences only once the potential wells at its surface are formed as a result of the charge rearrangement that is taking place upon the creation of junctions, i.e., upon contact with the other lead. Only then the following inequality is established:
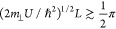
3where *ℏ* is the Planck constant. The calculated energies of the quantized states formed in the potential wells are shown in Figure 3a for each molecular layer. The details of the calculation at zero bias are described in the SI. A good agreement is observed between this calculation and the low bias conductance peaks in Figure 1d. In the energy range spanned by ∂*f*∂*E* at room temperature (see eq 1), three discrete levels within the potential wells are observed (in the positive bias polarity). The origin for only two observed levels in the reverse polarity and the fact that their position is not symmetric around zero bias is due to the complex distribution of the applied bias across the junction due to the space charge region.^49^ A self-consistent calculation, which considers this effect along with the deformation of the potential wells under bias and consequently the changes in the quantized states and their occupation deserves an elaborate treatment that is beyond the scope of this study.

**Figure 3:**
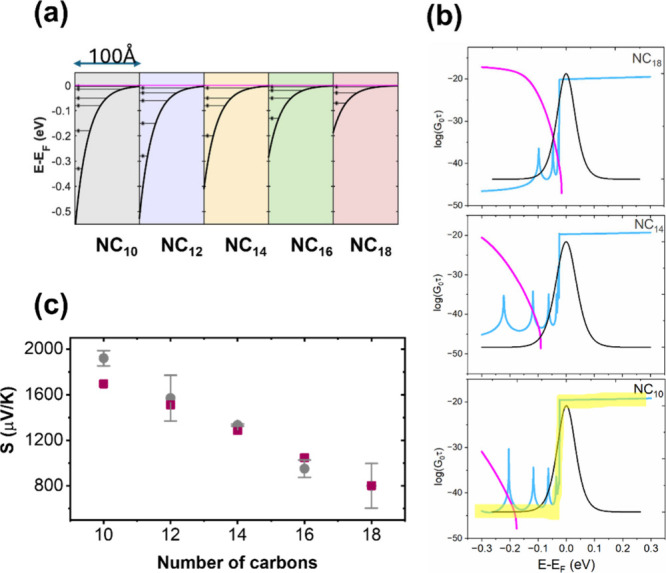
Quantization of the DOS within the potential wells and its effect on transmission and Seebeck. (a) Calculated built-in potentials of the formed accumulation layers for the different molecules. The horizontal lines mark the calculated quantized levels within each potential well. (b) Calculated transmission (multiplied by the quantum of conductance, *G*_0_) of junctions with the indicated molecules. The transmission is calculated separately for the conduction (cyan) and valence (magenta) bands. Also shown in each plot is the behavior of ∂*f*∂*E* at room temperature. Overlayed on the curves for NC_10_ is a step function (yellow), which represents an ideal transmission behavior for maximal |*S*|. Additional transmission curves appear in the SI. (c) A comparison between the experimental (gray) and calculated (dark red) Seebeck values of the different junctions.

A direct outcome of the established built-in potential within the Bi lead is also a shift of the molecular levels that accompanies the shift of the valence and conduction bands that is shown in Figure 2d.^50^ This process is rooted in the renormalization of the molecular levels. It is well established^57,58^ that the HOMO–LUMO gaps of molecules in the gas phase decrease upon adsorption to surfaces and that the magnitude of this change is proportional to the local DOS at the surface. Thus, for example, a metal induces a larger decrease of the gap than a semiconductor. As mentioned above, Bi is characterized by a low DOS in the vicinity of its Fermi level. As a result of the downshift of levels, the HOMO becomes coupled to a lower DOS and therefore experiences a smaller renormalization, i.e., a shift to lower energies (closer to the gas phase position). Figure 2a shows (in red) the calculated new energy position of the HOMO levels after renormalization. The details of this calculation are in the SI. Renormalization also causes the LUMO to be coupled to a larger DOS and hence experiences a larger change, which means a shift to lower energies. Since renormalization is estimated to decrease the HOMO–LUMO gap of molecules mostly by ∼3 eV,^57^ it is expected to change this gap for the alkane amines chains at the most from its initial value of ∼8 eV to ∼5 eV. Considering the above determined initial position of the HOMO levels (Figure 2a), after energy rearrangement, both the HOMO and the LUMO are expected to be sufficiently remote from the Fermi level to affect any substantial changes in the low bias transport properties. The enhancement of |S| and as will be shown below the unique conductance behavior of the junctions as a function of *n*, result from changes in Δ.

The transmission properties of the junctions were calculated by the nonequilibrium Green function formalism and the details of this calculations can be found in the SI. Figure 3b plots the calculated transmission curves for three different junctions, separating between the contribution of the conduction (cyan) and the valence (magenta) bands. Each plot also shows the curve of ∂*f*/∂*E* calculated at room temperature, which defines the energy window that determines the Seebeck (see eq 1). In the plot for the NC_10_ junction, a step-like feature (in yellow) has been added as a guide to the eye to better appreciate the progressive behavior in the different junctions. The step function is added since considering eq 1, a transmission curve in this form located at the Fermi level is expected to fully maximize |*S*|.^59^Figure 3b shows a progressive change of the overall transmission of the junctions toward this ideal step-function shape. As the molecules become shorter and the potential wells become deeper, two processes occur: (a) the quantized states within the conduction band are pushed to lower values and the energy gaps between them increase and (b) the valence band is shifted to lower energy values. Both processes effectively and progressively push Bi electronic states located below the Fermi level and that also participate in transmission through the junctions out of the ∂*f*/∂*E* window. In addition, due to the confining built-in potential, the contribution to tunneling below the Fermi level is substantially suppressed (note the logarithmic scale). The summation of all these effects culminates in efficient breaking of the electron/hole transport symmetry through the junctions and high |*S*| values. The first support for the model can be found in Figure 3c, which portrays a very good quantitative agreement between the calculated and measured |*S*| values.

Figure 4 gives further support to the above picture of progressive shift of levels. The conductance histograms shown in Figure 4a, were calculated by linear fitting of *I*–*V* curves in the ±0.1 V range. Their mean values, plotted in Figure 4b as a function of *n*, are in excellent agreement with the calculated values. Typically, in junctions with alkane molecules attached to metal leads, since the size of the HOMO–LUMO gap is length independent, the conductance is observed to decrease monotonically with *n,* with an exp(−β*n*) behavior^48^ and with a typical value of β ≈ 1 *n*^–1^. In contrast, Figure 4b shows an apparent exponential increase of the conductance for *n* ≤ 14. The origin for this is another proof for the dependency of the interfacial DOS of Bi on *n*. The exp(−β*n*) behavior corresponds to wide band conditions, i.e., when the DOS within the leads is constant and in eq 2 essentially defines θ = |*G*_m_|^2^. Since the DOS within the EGaIn is constant, the corresponding Δ of this lead is also constant. The peculiar behavior of conductance as a function of *n* results from the way both DOS_Bi_ (which affects Δ_Bi_) and *G*_m_ are changing with *n*. This is plotted in Figure 4c. As *n* becomes smaller, θ increases exponentially, but at the same time since *U* becomes larger (see Figure 3a) the effective integrated value of DOS_Bi_ (within the applied potential range) becomes exponentially smaller as the levels are shifted more out of the ∂*f*/∂*E* ≠ 0 window. Since the absolute change of DOS_Bi_ is larger than the change in θ, an overall increase in conductance is attained for *n* ≤ 14. Beyond this length the change in DOS_Bi_ is effectively negligible and then the exponential decrease of θ with *n* dominates.

**Figure 4:**
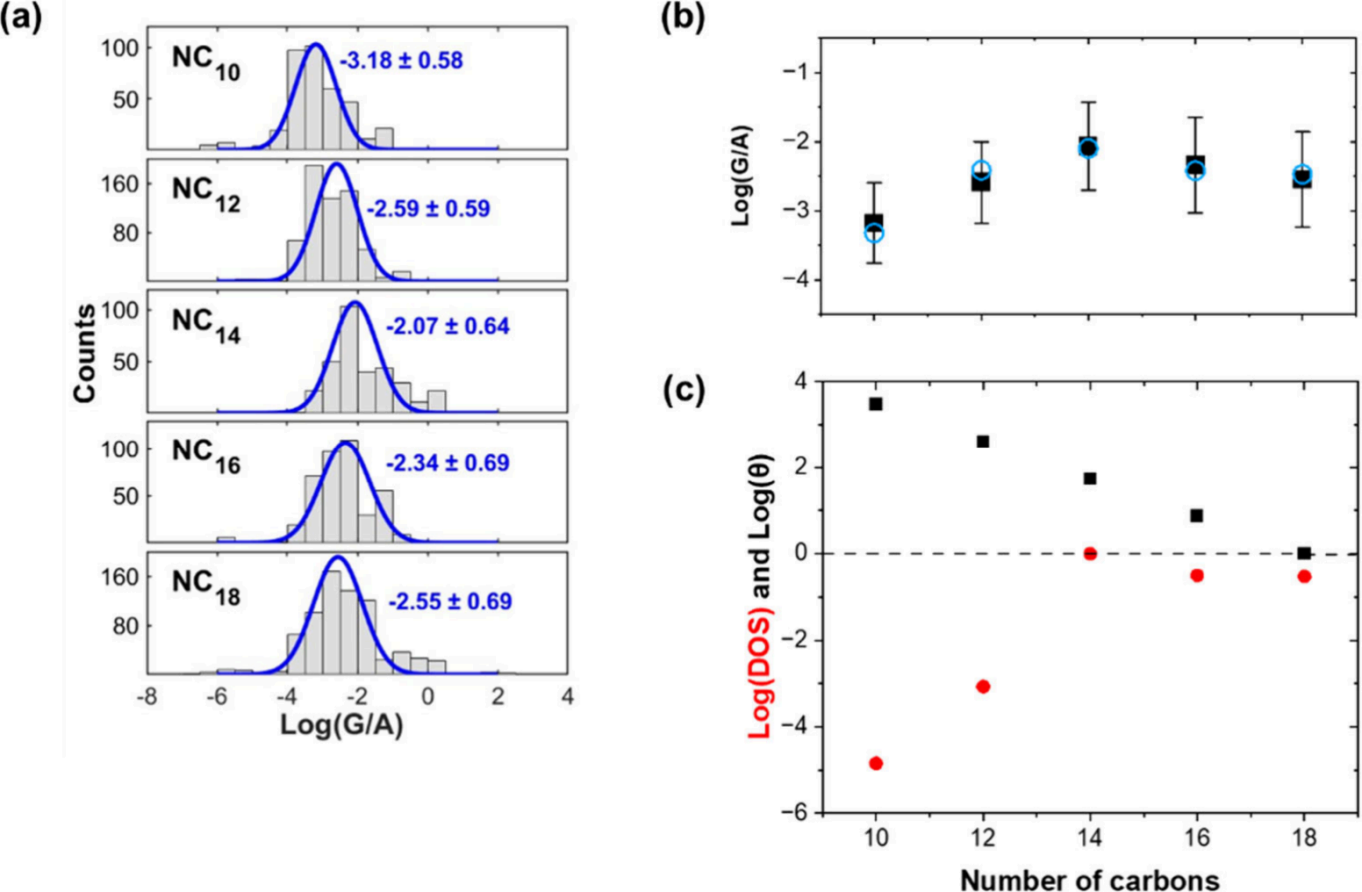
Unusual conductance behavior as a function of molecular length. (a) Histograms of area-normalized conductance measurements calculated for each junction by linear-fitting of *I*–*V* curves in the voltage bias range of ±0.1 V. (b) The mean values of conductance and their standard deviation as a function of molecular length (black rectangles) along with the calculated values (blue circles). In both (a) and (b) the area of the junctions, *A*, is in units of cm^2^. (c) A logarithmic plot of the change of transmission (black) and integrated (in an energy range of ±0.1 eV around the Fermi) DOS (red) as a function of molecular length. For clarity, the values are normalized in the case of transmission to the value for NC_18_ and in the case of DOS to the value for NC_14_.

To conclude, the method presented here enables the formation of molecular TE junctions with very high Seebeck values as a result of molecular manipulation and quantization of interfacial DOS. A short discussion on the power factor of the junctions appears in the SI. It essentially offers a glimpse into the potentially reach playground of various effects that molecular layers ensembled on semimetals can induce. Future work, for example, needs to explore the combination of the effects described here with conjugated molecules with small HOMO–LUMO gaps. The implications of this method as a tool to form 2D quantized states at interfaces of bulk semimetals also deserve further attention.

## Supporting Information Available

Additional experimental and characterization details and calculation methods of the built-in potentials and transmission functions (PDF)
