# Supplementary material for: Unusually High Thermopower in Molecular Junctions from Molecularly Induced Quantized States in Their Semimetal Leads
Source: Nano Lett. 2025 Feb 7;25(7):2756–62. doi: 10.1021/acs.nanolett.4c05852 (PMC11849033; doi:10.1021/acs.nanolett.4c05852)
Supplement: Supplementary file 1 — nl4c05852_si_001.pdf [file nl4c05852_si_001.pdf]

Supporting Information

Unusually high thermopower in molecular junctions from molecularly induced quantized states in their semimetal leads

Mor Cohen Jungerman[1], Shachar Shmueli[1], Pini Shekhter[2], Yoram Selzer[1]*

[1]School of Chemistry, Tel Aviv University, Tel Aviv 69978, Israel.

[2]The Tel Aviv Center for Nanoscience and Nanotechnology, Tel Aviv 69978, Israel.

***Formation and initial characterization of monolayers*.**

The 60nm thick, template-stripped Bi films used as the bottom lead in all junctions were prepared as described previously[1,2]. Formation of monolayers, devoid of any remnants of native oxide of Bi, requires an overnight assembly from ethanol solutions of the various amino alkanes under dry conditions inside a glovebox. As determined by contact angle measurements of water drops and similarly to monolayers of alkanethiols, only monolayers with chain length of $n \geq 10$, can be considered well-packed and ordered layers (top panel in Fig. S1a). Each of these monolayers was prepared by assembly from 100mM solutions of the relevant molecule. The bottom panel in Fig. S1a demonstrates based on contact angle measurements how the quality of, in this case, a NC_{14} monolayer depends on the concentration of the assembly solution.

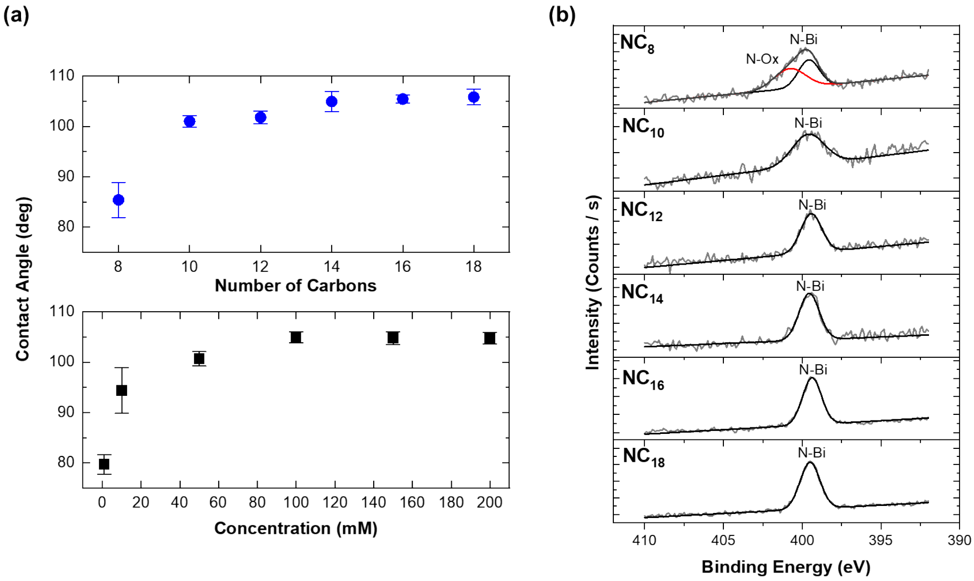

Figure S1. (a) Top panel: Contact angle of water on amino-alkane monolayers on Bi Bottom panel: contact angle of water on NC_{14} layers on Bi formed from solutions with the indicated concentrations. **(b)**XPS in the N_{1s} regime of the amino- alkane monolayers. Chains should be with n>8 for total removal of the oxide leaving only Bi-N bonds on the surface.

The uniformity of the layers was also determined by XPS measurements to verify the removal of native oxide and the exchange of oxygen-bonded Bi by amine-bonded Bi on the surface. For this purpose, XPS measurements were collected in the 1S regime of the amine. Fig. S1b shows that indeed for an assembly concentration of 100mM and n>8 there are no Bi-O-N bonds on the surface and at all surface sites Bi is directly connected to the amines (Bi-N).

Thermovoltage, I-V and conductance measurements

The experimental setup (Fig. S2a) involved an aluminum stage with a heating element that was gradually heated to the desired temperature upon which the sample was placed. To ensure good thermal contact between samples and stage, a thermal grease was applied to the back of the samples before mounting. The stage is much larger than all samples. This ensures that once the temperature of the stage is stabilized, it is also constant and uniform on the sample as well. Before all measurements, the position of the thermocouple was varied between different positions on the stage including also on a sample to verify constant temperature under heating conditions. The length of the drill which accommodates the heating unit and the position of the heating unit in the drilled tunnel was changed until a uniform temperature across the heating stage was reached also on top of a sample. From this point onward the thermocouple was placed right next to the

measured samples. We allowed the system to reach thermal equilibrium of at least 300 seconds after each temperature change. The entire setup of the heating stage, sample and EGaIn syringe were constantly kept under a blanket of Ar.

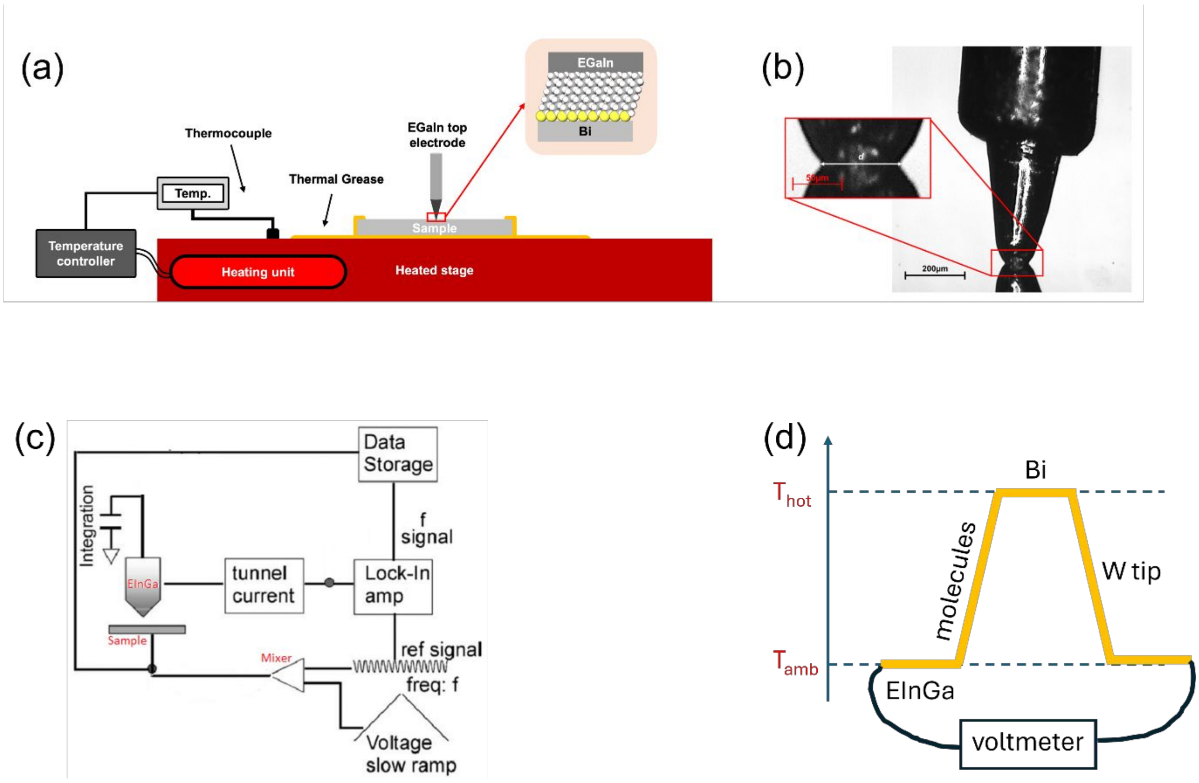

Figure S2. (a) A scheme of the experimental setup. (b) A high magnification image of a junction. (c) The lock-in setup used for the conductance measurements of the junctions. (d) The distribution of temperature in the setup.

Data acquisition was according to the following protocol: ~100 data points were collected per junction and at least 10 junctions were measured in different places per sample at a certain temperature differential. Prior to each measurement, the EGaIn tip was lifted to reach thermal equilibrium with the ambient surroundings. The temperature of the tip was assumed to be at room temperature, see discussion below. To confirm contact and the formation of the desired junction, I-V measurements were conducted before and after each thermovoltage measurement. The size of each contact was determined by a telescope connected to a camera which allows us to measure the diameter of the contacts at high magnification (Fig. S2b). Each EGaIn conical tip was used for not more than 10 junctions to minimize the effect of surface contamination of the tip. In this way the measurements reflect trace-to-trace, junction-to-junction, tip-to-tip and sample-to-sample variations, but avoid overweighting one or some of them. The yield of working junctions was 60%. The histograms of ΔV were fit to single Gaussian curves, from which the mean value (ΔV) and standard deviation ($\sigma_{\Delta V}$) were extracted.

The thermovoltage, resulting from the temperature gradient between the bottom Bi film and the top EGaIn lead was measured by a voltmeter (Keithley 2000) with an input impedance in the relevant range of >10GΩ. I-V curves in the range of ±0.1V were measured with a Keithley 2400 source-meter. Figure S2c shows schematically the setup used for the conductance measurements shown in figure 1D. We used a homebuilt mixer to combine the linear voltage ramp and the *ac* signal (with a frequency of 137Hz and an amplitude of few mV) from the lock-in.

Fig. S2d shows the temperature distribution on all the components of the experimental system. As the temperature across the Bi surface is uniform (see discussion above) there is no thermovoltage across the sample. The figure also shows no temperature gradient on the EGaIn drop. See a discussion on this point in a detailed section below. The Bi film is connected to the voltmeter by a Tungsten tip. As the Seebeck of this material is 1μV/K, the highest thermovoltage that is developed across this tip is 7μV (for $\Delta T = T_{hot} - T_{amb} = 7K$). This value is negligible relative to the thermovoltage across the junctions. The reported overall thermovoltage is: $\Delta V = -(S_{molecules} - S_{W\ tip})(T_{hot} - T_{amb})$.

Temperature gradient across the EGaIn drop.

Former Seebeck measurements of molecular junction employing STM or AFM tips as a top contact[3] assume that the temperature gradient between the bottom substrate and the tip, *ΔT,* falls entirely on the molecules, i.e., that the tip remains at ambient temperature (T_{amb}). This claim is supported by thermal transport simulations, which suggest an uncertainty of 5% in this claim. Former Seebeck measurements of EGaIn/molecules/Au junctions (ref. 42 in the main text) claim that albeit obvious changes in geometry and thermal conductivity versus the former STM/AFM experiments, these changes are not sufficient to change the <5% error in *ΔT.*

We have verified this claim by numerically calculating the thermal transport in the setup. The parameters used in the calculations are shown in figure S3a.

The equation to be solved numerically is:

(S1) $$-kA_{j-1,j}\frac{T_j - T_{j-1}}{dy} + kA_{j,j+1}\frac{T_{j+1} - T_j}{dy} - h_{conv}pdy\left(T_j - T_{amp}\right) = \rho c A_j \frac{\partial T}{\partial t} dy$$

with the following boundary conditions:

(S1a) $T(y \rightarrow \infty, t) = T_{amb}$

(S1b) For T(y=0,t): $k\frac{T_1-T_0}{dy} - h_{mol}(T_0 - T_{hot}) = \rho c\frac{\partial T}{\partial t}dy$

The first expression on the left side of equation S1 describes the energy flux at the bottom side of a certain layer with an index j in the EGaIn electrode. Similarly, the second expression on the left side describes the energy flux at the top side of the same layer.

Note that due to the shape of the EGaIn drop, see image in figure S2, the bottom and top cross sections of a layer are not identical: $A_{j-1,j} \neq A_{j,j+1}$. These cross sections depend on the distance y, measured from the interface EInGa/molecules in the following way:

(S2) $$A(y) = \pi(R_{junc} + {}^{y}/_{tan\theta})^2$$

The third expression on the left side of equation S1 describes energy flux by air convection through the perimeter area of each layer, $pdy = 2\pi\left(R_{junc} + {}^{y}/_{tan\theta}\right)dy$.

In all simulations, T_{amb}=300K. Based on images of the EGaIn drop, such as the one shown in figure S2b, the angle varies in the range: $\theta =$ 60^0-70^0. R_{junc} was taken to be 40μm. Varying h_{conv} within a reasonable value range of 10-100 W/m^{2}K, appears not to affect the results of the calculations. This can be rationalized by the large ratio between the volume of the EGaIn drop and its surface area.

In the expression on the right side of equation S1, ρ (6.25 g/cm^3) and c (300 J/Kg K) are the density and heat capacity of EInGa, respectively.

In the second boundary condition (equation S1b) the thermal conduction through the molecules, h_{mol}, is estimated to be ~10^6 W K^{-1} m^{-2}, considering a typical thermal conductance of 30 pW K^{-1} per molecule and a packing density of 0.21 nm^{-2}. T_0 is the temperature on the Bi side, which in the experiments was not more than 7K above T_{amb}.

The calculated change of temperature, $T - T_{amb}$, as a function of distance from the interface along the dashed red line in figure S3b, is plotted in figure S3c. The plot shows that indeed, the temperature at the interface within the EInGa drop remains at T_{amb}.

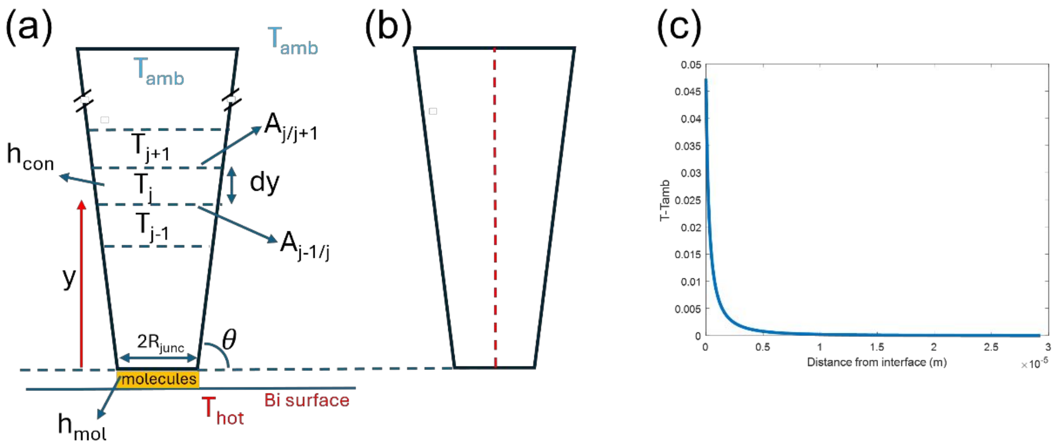

Figure S3. (a) A sketch illustrating the nomenclature used in the numerical analysis of the heat conduction within a junction. (b) and (c) The temperature shown in (c) is calculated along the red dashed line in (b).

Calculation of the space-charge region in Bi

We perform the calculation classically, avoiding iterations considering that charge within this region is in quantized levels. The calculation is based on a one-dimensional Poisson's equation for a one-electron potential energy *u* given by:

(S3) $$\frac{d^2u}{dx^2} = \frac{4\pi e^2}{\epsilon_{Bi}}[n_e(x) - n_h(x)]$$

where ϵ_{Bi} is the permittivity of Bi ($\epsilon_{Bi} = 100$). The quantities $n_e(x)$ and $n_h(x)$ are the particle densities of electrons and holes, respectively and we assume that Bi extends from $x = 0$ to $x = \infty$.

The boundary conditions of eq. S1 are:

(S4a) $$\lim_{x\to\infty} u(x) = 0$$

(S4b) $$\lim_{x\to\infty} du/dx = 0$$

At zero temperature the particles densities are given by the Fermi-Thomas model to be:

(S5a) $$n_e(x) = n_0\left[1 - \frac{u}{\xi_e}\right]^{\frac{3}{2}}\theta(\xi_e - u)$$

(S5b) $$n_h(x) = n_0\left[1 - \frac{u}{\xi_h}\right]^{\frac{3}{2}}\theta(|\xi_h| - u)$$

where n_0 is the bulk density of electrons and holes (3.5×10^{17} cm^{-3}) and ξ_e (=22meV) and ξ_h (=12meV) are respectively their Fermi energies.

Using

(S6) $$\theta(x) = 1\ ; x > 0$$

$$=0\ ;\ \ x < 0$$

eq. S1 can be reduced to:

(S7a) $$x = \left(\frac{5\epsilon_{Bi}}{16\pi n_0 e^2}\right)^{1/2}\int_0^{u(x)} du/D(u)$$

(S7b) $$D(U) = \left[\zeta_h\left[1 + \frac{U}{\zeta_h}\right]^{\frac{5}{2}}\theta\left(1 + \frac{U}{\zeta_h}\right) + \zeta_e\left[1 - \frac{U}{\zeta_e}\right]^{\frac{5}{2}}\theta\left(1 - \frac{U}{\zeta_e}\right) - (\zeta_h + \zeta_e)\right]^{\frac{1}{2}}$$

The quantity u(0) is calculated assuming that $\Delta\varphi$, the difference between the work functions of the metal (EGaIn, 4.15eV) and Bi (4.3+ΔWF) is divided between a molecular layer of thickness x_{mol} from $x = -x_{mol}$ to $x = 0$ and the semi-infinite Bi. The boundary conditions at the molecular layer/Bi interface are:

(S8a) $$u(x\rightarrow0_{mol}) = u(x\rightarrow0_{Bi})$$

(S8b) $$\epsilon_{mol}u'(x\rightarrow0_{mol}) = \epsilon_{Bi}u'(x\rightarrow0_{Bi})$$ (using $\epsilon_L = 2$).

(S8c) $$u(x) = F(x + x_{mol}),\quad -x_{mol} \leq x \leq 0$$

(S8d) $$\Delta\varphi = -u(0) + Fx_{mol}$$

where *F* is the field across the molecular layer.

By taking the derivative of eq. S7a and S8c, we can eliminate *F* and obtain the following set of equations:

(S9a) $$U \equiv u(0) = -\Delta\varphi + \epsilon_R u'(0)x_{mol}/\epsilon_L$$

(S9b) $$u'(0) = -\left(\frac{16\pi n_0 e^2}{5\epsilon_R}\right)^{\frac{1}{2}} D(U)$$

which are then solved iteratively.

The formed built-in potential is then:

(S10a) $$u(x) = Uexp(-x/L)$$

with a space-charge length, L, defined as:

(S10b) $$L = \left[\frac{6\pi n_0 e^2}{\varepsilon_{Bi}\varepsilon_0}\left(\frac{1}{\zeta_h} + \frac{1}{\zeta_e}\right)\right]^{-\frac{1}{2}} \sim 100\text{Å}$$

where ε_0 is the permittivity of vacuum.

Calculation of the transmission function.

To simplify the calculation, we treat the studied systems as 1D structures. The apparent agreement between the calculations and the measured Seebeck and conductance justify the assumption. The physical reasoning behind it is shown in Fig. S4. The intrinsic potential step between the Bi and the EGaIn electrodes affects the electrons crossing between them in two ways: An electron's momentum perpendicular to the step must be greater than a critical value k_c to pass into the Bi and any electron crossing into the EGaIn gains perpendicular momentum at the step. An electron in the EGaIn must have $k_x > k_c$ momentum, where the latter is defined in Fig. S4, to cross the interface. Since the difference between the two Fermi energies is large, k_c and k_f (the Fermi momentum) are similar in size. Thus, the transporting electrons in the EGaIn hardly have any momentum parallel to the interface and therefore behave similarly to within a 1D structure. At the same time within the Bi, the electrons confined perpendicular to the interface within a potential well of 10nm in width, hardly have any time to spread out parallel to interface once they bounce from the confining potential which causes them to move back towards the interface. The reason for this behavior is the large anisotropy in the effective mass of the electrons in Bi. The mass of electrons moving perpendicular to the interface in the (111) direction is smaller by an order of magnitude compared to other directions. All this results in an effective Fermi surface on the Bi side that is inhomogeneous with a 1D-like structure.

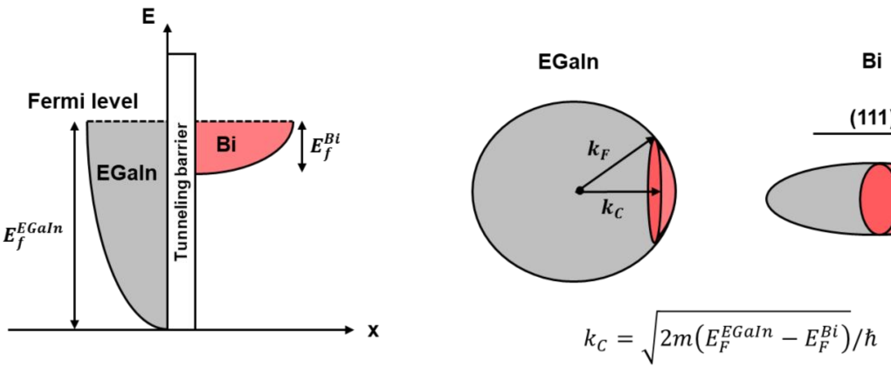

Figure S4. The interface between EGaIn and Bi.

The transmission of the junctions was calculated using the non-equilibrium Green function formalism. The approximated band structure of Bi and molecular levels used for this calculation are shown in Fig. S5a. The transmission is calculated for each band of the leads separately, as their dispersion and hence their surface Green functions have different values at each energy. We ignore in this calculation that the two involved bands are located at different momentum in k-space within the dispersion of Bi. As both the HOMO and LUMO of the amino-alkane chains are localized on the amino group (Fig. S5b), only two levels represent each molecule with a coupling value to the EGaIn lead that is attenuated exponentially with a factor of exp(-βn) using β=1.

The Hamiltonian of the molecule is:

(S11) $$H = \begin{pmatrix} \varepsilon_1 & t \\ t & \varepsilon_2 \end{pmatrix}$$

and the corresponding (retarded) Green function is:

(S12) $$G^R(E) = \frac{1}{(E+i\Gamma_L-\varepsilon_1)(E+i\Gamma_R-\varepsilon_2)-t^2}\begin{pmatrix} E + i\Gamma_R - \varepsilon_2 & t \\ t & E + i\Gamma_L - \varepsilon_1 \end{pmatrix}$$

The coupling between molecular sites is t=0.7eV and the energy of the two levels are: ε_1= 1.0eV, ε_2=-1.0eV. This results in molecular electronic level at -1.7eV below the Fermi level, in agreement with the (average) position of the HOMO based on the UPS measurements.

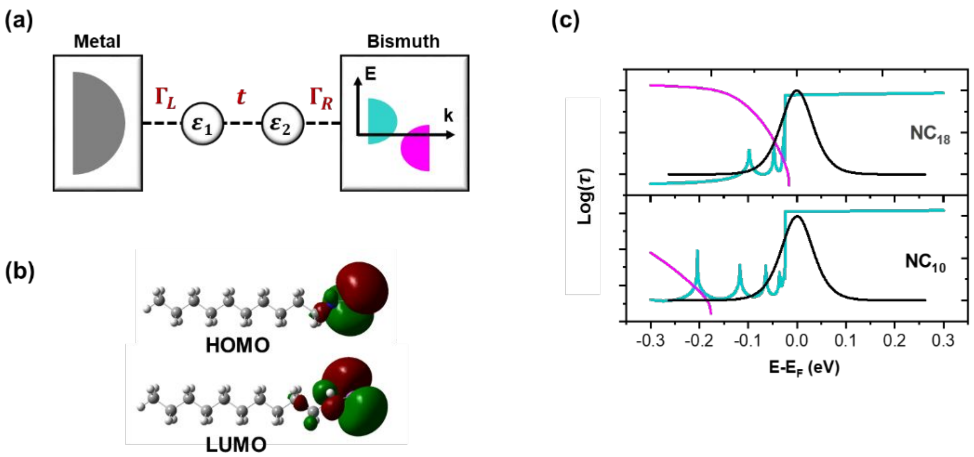

Figure S5. Model and theoretical results. (a) The model and parameters used for the calculation of transmission and Seebeck. **(b)** The HOMO and LUMO of aminoalkanes connected to Bi are localized on the amine group. **(c)** Calculated transmission (in log scale) of NC_{10} and NC_{18} junctions, showing the contribution of the electrons (cyan) and holes (magemta) bands. In dashed green is $\frac{\partial f}{\partial E}$, calculated at T=300K.

Assuming wide band conditions on the EGaIn side, the coupling of the left molecular site to this lead does not depend on energy and is taken here to be Γ_L=5meV. In contrast, the coupling to the Bi lead, $\Gamma_R(E)$, is energy dependent and depends on the surface green function of this lead, G_S^R (E), which due to the built-in potential depends on the attached molecule.

Using a base coupling value for the Bi lead: Γ_R^0=5meV, the self-energy is then:

(S13)
$$\Sigma_S^R(E) = \begin{pmatrix} 0 & 0 \\ 0 & \Gamma_R^0 G_S^R(E)\Gamma_R^0 \end{pmatrix}$$

and accordingly, Γ_R, used in eq. S11, is:

(S14)
$$\Gamma_R(E) = i(\Sigma_S^R(E) - \Sigma_S^A(E))$$

The calculation of the surface Green function, G_S^R , proceeds in the following way: We first calculate the Hamiltonian of the Bi lead. Since we treat the leads as a 1D problem, we find that N=600 grid points (assumed to be separated by a lattice constant *a*=2Å in the (111) direction[4]) is sufficient to reproduce the experimental results without the need to perform the calculation iteratively. Note, that as shown in the main text, L is in the order of ~100 Å, much smaller than the size of the Bi lead considered by the calculation.

The Bi Hamiltonian is:

(S15)

$$H_{Bi} = \begin{pmatrix} \alpha+V_0 & -\gamma & 0 & & & & \\ -\gamma & \alpha+V_1 & -\gamma & \cdots & & 0 & \\ 0 & -\gamma & \alpha+V_2 & & & & \\ & \vdots & & \ddots & & \vdots & \\ & & & & \alpha+V_{N-2} & -\gamma & 0 \\ & 0 & & \cdots & -\gamma & \alpha+V_{N-1} & -\gamma \\ & & & & 0 & -\gamma & \alpha+V_N \end{pmatrix}$$

where α=0 and $V_j = -Uexp(-a \cdot j/L)$, where *j* is a site index. As discussed above, $U = u(0)$, depends on the molecule.

The eigen values of this Hamiltonian give the quantized levels formed within the potential well at the interface and are plotted in Fig. 3a.

With a coupling of γ between the sites in Bi, the dispersion of each band is assumed to be $E = \varepsilon + 2\gamma coska$. We ignore the fact that for the electrons at the L point the dispersion is according to the Lax model[5], which assumes large electron-hole interaction. Yet, for the purpose of Seebeck calculation, close to the Fermi level, the Lax model can be accurately described by a parabolic dispersion[6]. Using a Taylor series to approximate the dispersion, we define γ to be: $\gamma = -\hbar^2/2ma^2$. As we treat the bands separately, we use $m_e = 0.05m_0$ and $m_h = 0.7m_0$ for the effective mass at the L and T points, respectively and therefore have $\gamma_{e/h}$.

Within the dispersion, the Fermi level is made to reside at ξ_e above the lower edge of the electron band and $\xi_h$below the upper edge of the hole bands.

For each energy, *E*, relatively to the edge of the corresponding band, the momentum value is calculated by:

(S16) $$ka = cos^{-1}(E/2\gamma_{e/h})$$

which can then be used to calculate Σ:

(S17) $$\Sigma = \begin{pmatrix} 0 & \cdots & 0 \\ \vdots & \ddots & \vdots \\ 0 & \cdots & \gamma\exp(-ika) \end{pmatrix}$$

Where Σ is an N×N matrix (with N=600) like H_{Bi}.

The Green function of the Bi lead is then:

(S18) $$G_R(E) = ((E + \eta i)I - H - \Sigma)^{-1}$$

with η being an infinitely small number and I is an identity matrix.

The surface Green function is $G_S^R = G_R(1,1)$ and can be used in eq. S13 to calculate the self-energy Σ_S^R, which can then be used to calculate Γ_R.

For each energy the transmission is calculated by:

(S19) $$T(E) = Tr(\Gamma_L G^R \Gamma_R G^A)$$

Fig. 3S reproduces some of the transmission curves shown in the main text.

The Seebeck behavior as a function of n for superexchange and gateway mechanisms.

In the main text we argue that the Seebeck in the measured systems, assuming a wide band approximation, should have an $|S| \propto n/(E_{\text{Molecular}} - E_F)$ behavior. Here we discuss the origin of this equation and consider an alternative transport mechanism, namely a gateway transport and show that it should also have the same Seebeck behavior as a function of *n*.

The conceptual picture for the deep-tunneling superexchange mechanism is shown in figure S6a.

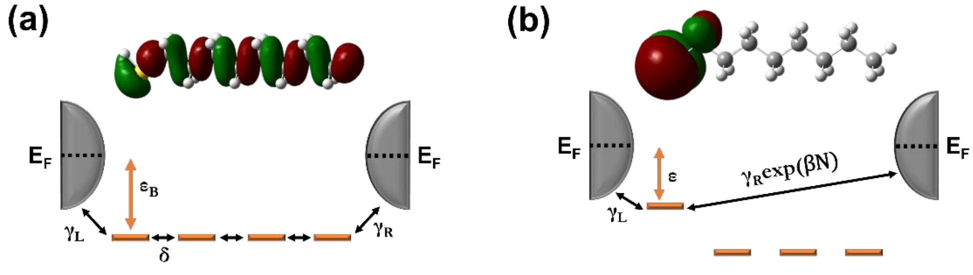

Figure S6. Schematic presentation of two transport mechanisms through amine-alkane chains. (a) Superexchange tunneling. (b) Gateway state localized near the left lead and with exponentially attenuated coupling to the right lead.

As reference 51 and 52 in the main text describe the transmission in this case decreases with the molecular length (n), according to:

(S20) $$\tau(E) = \left(\frac{\delta}{E - E_{molecule}}\right)^{2n} \frac{\gamma_L \gamma_R}{\delta^2}$$

where δ is the coupling between sites, γ_L and γ_R are the coupling between the end sites to the left and right leads, respectively.

As a result of this exponential decay in transmission, the Seebeck coefficient (S), calculated for E=E_F, should increase linearly with molecular length, n, according to the Mott formula (on the right side of equation 1 in the main text). This gives:

(S21) $$S = \frac{\pi^2 k_B}{3e} \frac{k_B T}{E_F - E_{molecule}} \times 2n$$

i.e., $|S| \propto n/(E_F - E_{\mathrm{molecule}})$

An alternative transport mechanism considers a gateway state associated with and localized at the metal-S bond[7-9] (Fig. S6b). Under such conditioned transmission can be approximated by:

(S22) $$T = \frac{1}{1 + \left(\frac{E-\varepsilon}{E_F-\varepsilon}\right)^2 \exp(\beta N)}$$

where ε is the energy of the gateway state and β is the distance-dependent tunneling attenuation factor from this state to the leads. Distance is measured also here as the number of carbon units.

Applying the Mott formula to this expression of transmission gives the following expression for the Seebeck coefficient:

(S23) $$S = \frac{\pi^2 k_B^2 T}{3e}\left[\frac{\partial \beta}{\partial E} N - \frac{2}{E_F - \varepsilon}\right]$$

The derivative $\frac{\partial \beta}{\partial E}$ behaves according to a superexchange mechanism for a charge localized on the gateway state that can tunnel to one lead under a potential defined by the coupled levels of the carbons in the chain, i.e., it also behaves according to $\propto n/(E_{\mathrm{Molecular}} - \varepsilon)$. Thus, in both mechanisms, (the absolute value of) S increases with n. Note that the contribution of the second term within the brackets to S is negligible.

A comparison between the effect of thiol and amino-alkanes on the properties of the built-in potentials within the Bi leads.

When using alkane-thiols as molecules in junctions with similar leads, a built-in potential within the Bi side of the junctions is also created[1]. However, since alkane-thiols *decrease* the work function of Bi, the built-in potential in the previous study was in the form of a depletion layer. All this is summarized in figure S7. A depletion-like potential, formed with alkane-thiols, pushes the levels at the interface up with a dominant behavior of the valence band at the Fermi level. In the case of amino-alkanes it is the conduction band that dominates the DOS at the Fermi level. This explains

why no quantization of levels has been observed when using alkane-thiols. The reason for this is the difference between the effective mass of the charge carriers in the valence and conductions bands, which are $0.6340m_0$ and $0.0052m_0$, respectively, where m_0 is the mass of a free electron. As explained in the main text quantization occurs only when the following condition is realized: $\left(2m_{\perp}U/\hbar^2\right)^{1/2}L \gtrsim \frac{1}{2}\pi$. Since both the length, *L*, and the depth of the potential well, *U,* in the accumulation and depletion layers are the same, quantization can occur only with the lighter charge carriers, i.e., within the conduction band and therefore only when amino-alkanes are used. Quantization of the valence band carriers necessitates L~1nm, which is most likely non-realistic.

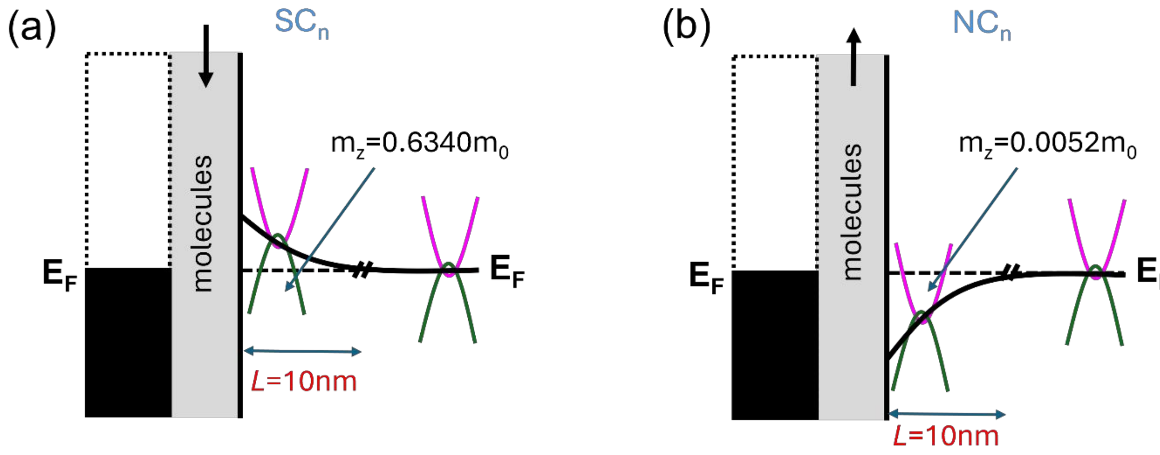

Figure S7. The difference between the effect of thiol and amino-alkanes on band alignment and quantization of levels. (a) Monolayers of alkane-thiols decrease the work function of Bi making, it smaller than the work function of EInGa, and as a result form a depletion-like layer within the Bi. Due to the shift of levels, the valence band aligns with the Fermi level. (b) Monolayers of amino-alkanes increase the work function of Bi and therefore result in an accumulation layer. Here, the conduction band is aligned with the Fermi level.

A short discussion concerning the power factor of the measured junctions.

The power factor of thermoelectric devices, defined as $PF=S^2G$ is an essential parameter, which mainly defines their efficiency as cooling elements. A recent review compiles the available information regarding this parameter for junctions based on molecular monolayers[10]. Indeed, we find that the *PF* in our junctions, in comparison to previous studies, is higher by a factor of 10^4. This can readily be explained for alkane-chains by the increase in the Seebeck values in our junctions by two orders of magnitude (from ~10μV/K to the 1mV/K range). Specifically, for

example, the *PF* for EGaIn/SC_{12}/Au junctions is 8×10^{-12} μW K^{-2} m^{-1}, while in our junctions with NC_{12} it is 2.56×10^{-8} μW K^{-2} m^{-1}.

Low bias conductance measurements

Room temperature conductance measurements, performed as described above by a lock-in technique, were conducted to resolve the quantized levels within the interfacial potential wells of the junctions. Figure 1d in the main text shows the results for NC_{10} and NC_{12} junctions.

In figure S8, we show additional measurements of junctions with the same molecules. They are presented here to emphasize several important points.

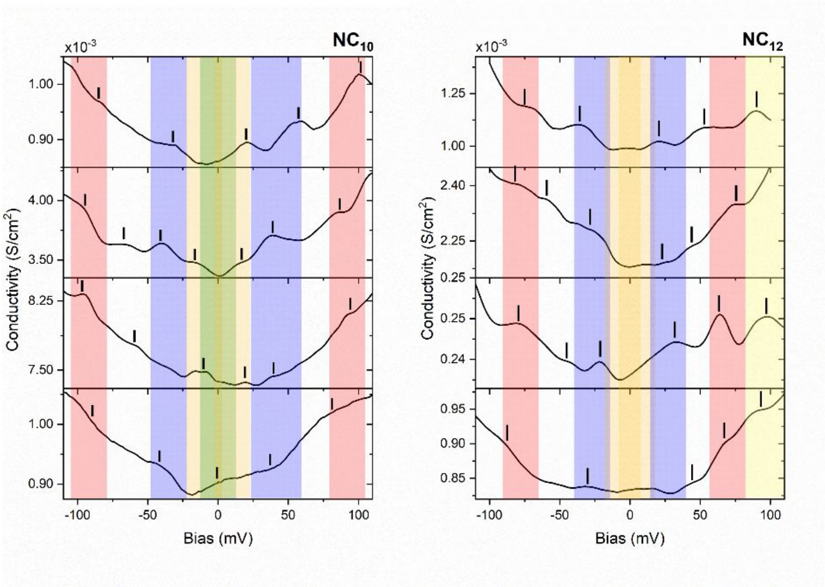

Figure S8. Additional examples of conductance measurements of NC_{10} and NC_{12} junctions.

The reproducibility of the peaks is quite high considering the size of the junctions and possible sources for variation between them. Still, some peaks in certain junctions are more difficult to resolve. See for example the peaks within the white zones between (-50mV)-(-80mV) in the NC_{10} measurements and between (-40mV)-(-60mV) in the results for NC_{12}. The origin for this could be variation between junctions but also because of limited detection capability resulting from the fact that the measurements are performed at room temperature with a resolution governed by thermal smearing at the Fermi energy.

We also note that the number of resolved peaks in both bias polarities are not in all cases identical. Generally, in all junctions, three peaks, i.e., three quantized levels in agreement with the

calculations, could be resolved in the positive bias. However, in negative polarity, in many junctions only two peaks can be observed. As mentioned in the main text, we attribute this to a different bias-polarity dependent response (change) of the built-in potential distribution across the junctions. When a bias is applied, the potential wells at the interface are changed and with them also the quantization of levels[1,2]. If the change in the form of the potential wells depends on the polarity of the applied bias, quantization becomes also polarity dependent[2]. This situation is very much different from STM probing of interfacial states, where most of the applied potential falls on the gap between the tip and sample, as then the effect of the applied bias and its polarity does not affect the (zero bias) DOS. In our system, most of the applied potential falls within the Bi leads[1,2] and therefore the form of the potential wells is very sensitive to the applied potential and its polarity.

Calculated conductance curves

The following graph summarizes the calculated conductance curves for all junctions. The plot on the bottom right was calculated for a junction without any built-in potential within the Bi.

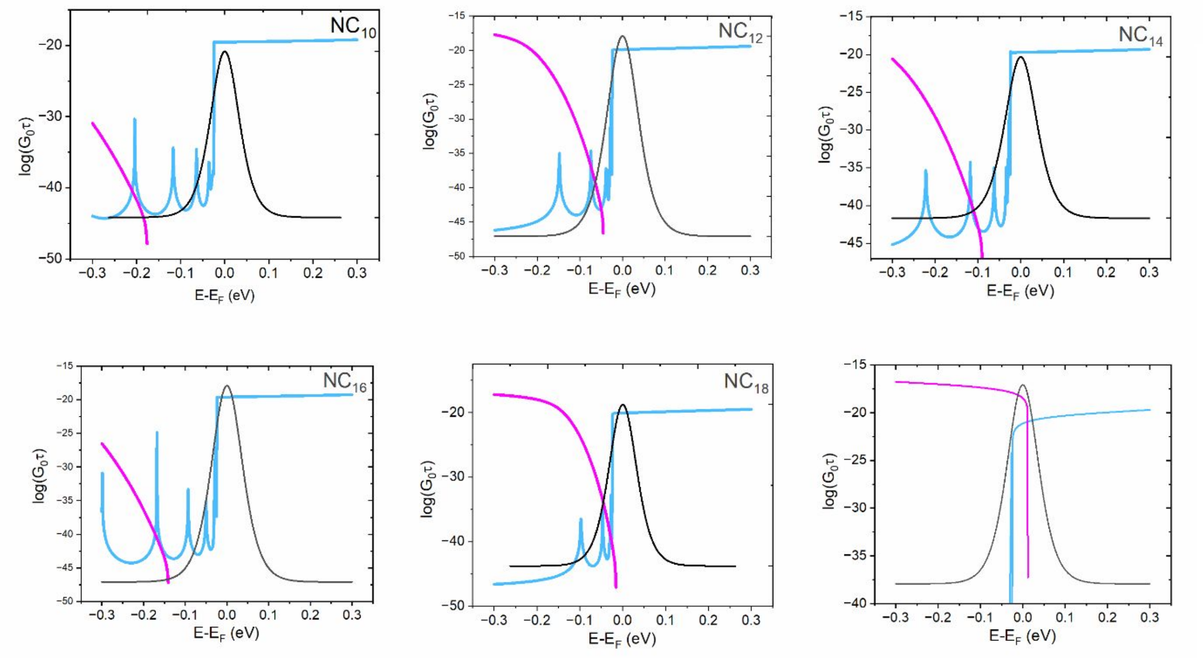

Figure S9. Calculated conductance curves.

References

(1) Frank, T.; Shmueli, S.; Cohen Jungerman, M.; Shekhter, P.; Selzer, Y. Large Seebeck Values in Metal-Molecule-Semimetal Junctions Attained by a Gateless Level-Alignment Method. *Nano Lett.* **2023**, 23, 10473.

(2) Shmueli, S.; Cohen Jungerman, M.; Shekhter, P.; Selzer, Y. Efficient Molecular Rectification in Metal-Molecules-Semimetal Junctions. *J. Phys. Chem. Lett.* **2024**, 15, 10602.

(3) Reddy, P.; Jang, S. Y.; Segalman, R. A.; Majumdar, A. Thermoelectricity in molecular junctions *Science* **2007**, 315, 1568.

(4) Liu, Y.; Allen, R. E. Electronic Structure of the Semimetals Bi and Sb. *Phys. Rev. B* **1995**, 52, 1566.

(5) Lax, B.; Mavroides, J. G. Cyclotron Resonance. *Solid State Phys.* **1960**, 11, 261.

(6) Lin, Y. M.; Sun, X.; Dresselhaus, M. Theoretical Investigation of Thermoelectric Transport Properties of Cylindrical Bi Nanowires. *Phys. Rev. B.* **2000**, 62, 4610.

(7) Li, C.; Pobelov, I.; Wandlowski, T.; Bagrets, A.; Arnold, A,; Evers, F. Charge transport in singe Au-alkandithiol-Au junctions. *J. Am. Chem. Soc.* **2008**, 130, 318.

(8) Huisman, R. H., Guedon, C. M.; van Wees, B. J.; van der Molen, S. Interpretation of transition voltage spectroscopy *Nano. Lett.* **2009**, 9, 3909.

(9) Xie, Z.; Baldea, I.; Frisbie, C. D. Energy Level Alignment in Molecular Tunnel Junctions by Transport and Spectroscopy: Self-Consistency for the Case of Alkyl Thiols and Dithiols on Ag, Au, and Pt Electrodes. *J. Am. Chem. Soc.* **2019**, 141, 18182.

(10) Park, S.; Kang, S.; Yoon, H. J. Power Factor of One Molecule Thick Films and Length Dependence *ACS. Cen. Sci.* **2019**, 5, 1975.
